# Protective Effects of Tamarillo (*Cyphomandra betacea*) Extract against High Fat Diet Induced Obesity in Sprague-Dawley Rats

**DOI:** 10.1155/2015/846041

**Published:** 2015-06-11

**Authors:** Noor Atiqah Aizan Abdul Kadir, Asmah Rahmat, Hawa Z. E. Jaafar

**Affiliations:** ^1^Department of Nutrition and Dietetics, Faculty of Medicine and Health Sciences, Universiti Putra Malaysia, 43400 Serdang, Selangor, Malaysia; ^2^Department of Crop Science, Faculty of Agriculture, University Putra Malaysia, 43400 Serdang, Selangor, Malaysia

## Abstract

This study aims to investigate the protective effect of *Cyphomandra betacea* in adult male Sprague-Dawley rats fed with high fat diet. Rats were fed on either normal chow or high fat diet for 10 weeks for obesity induction phase and subsequently received *C. betacea* extract at low dose (150 mg kg^−1^), medium dose (200 mg kg^−1^), or high dose (300 mg kg^−1^) or placebo via oral gavages for another 7 weeks for treatment phase. Treatment of obese rats with *C. betacea* extracts led to a significant decrease in total cholesterol and significant increase in HDL-C (*p* < 0.05). Also there was a trend of positive reduction in blood glucose, triglyceride, and LDL-C with positive reduction of body weight detected in medium and high dosage of *C. betacea* extract. Interestingly, *C. betacea* treated rats showed positive improvement of superoxide dismutase (SOD) activity and glutathione peroxidase (GPx) activity along with a significant increase of total antioxidant status (TAS) (*p* < 0.05). Further, rats treated with *C. betacea* show significantly lower in TNF-*α* and IL-6 activities (*p* < 0.05). This study demonstrates the potential use of *Cyphomandra betacea* extract for weight maintenance and complimentary therapy to suppress some obesity complication signs.

## 1. Introduction

Obesity is currently the most common metabolic disease in the world; it is significantly associated with potentially life-threatening comorbidities. Either obesity itself or comorbidities of obesity are responsible for increased cardiovascular risk. Obesity is associated with most of the components of metabolic syndrome, the leading cause of type 2 diabetes. The comorbidities of obesity and type 2 diabetes associated with insulin-resistance syndrome include obstructive sleep apnea, hypertension, polycystic ovary syndrome, nonalcoholic fatty liver disease, and certain forms of cancer [1].

When obesity persists for a long time, therefore, the antioxidant sources can be depleted, decreasing the activity of enzymes such as superoxide dismutase (SOD) and catalase (CAT) [2]. The activity of SOD and glutathione peroxidase (GPx) in individuals with obesity is significantly lower compared with that in healthy persons, having implications for the development of obesity-related health problems [3, 4].

Obesity is considered as a low-grade chronic inflammation. The levels of proinflammatory adipokines and protein such as tumour necrosis factor-*α* (TNF-*α*), interleukin (IL-6), and inducible nitric oxide synthase (iNOS) in adipose tissues and C-reactive protein (CRP) in plasma are increased in obese people [5]. The uncontrolled inflammatory response leads to a persistent proinflammatory state resulting in rising blood pressure, thrombosis, dyslipidaemia, and metabolic disease in obesity [6–8].

High fat diet induced obese rat model has been considered as a popular preference for its ability to mimic the usual way of obesity in human. High fat diet is one of the major factors causing obesity and long term intake of high fat diet showed significant increase in abdominal fat weight in mammals [9]. Woods et al. [10] reported that in high fat diet induced obese rat model, the high fat rats weighed more than control rats. In addition, they also developed subsequently more adipose tissue than control rats and acquired the insulin resistance and hyperleptinemia typically associated with obesity.

Interestingly, eating fruits and vegetables can ensure the adequate supply of micronutrient, dietary fibres, and phytochemicals which in turn maintain the body in healthy state [11]. The rationale behind the potential role of fruit in the prevention of overweight and obesity is related to special features of whole fruit which include high water content, low energy density, and high content of dietary fibres, of which viscous dietary fibres constitute a considerable proportion [12].

In Malaysia,* Cyphomandra betacea* is locally known with various names such as “Buah Cinta,” “Moginiwang,” “Pokok Tomato,” or “Tamarillo” and cultivated in Cameron Highland and Kundasang, Malaysia [13].* C. betacea* are considerably nutritious because of their high vitamin content.* C. betacea* was demonstrated to contain phytochemicals including beta-carotene anthocyanins, flavonols, phenolic acids, and large amounts of ascorbic acid [14–18]. To date,* C. betacea* remains unexplored except for its antioxidant profile and, to the best of our knowledge, this is the first study to evaluate the protective effects of* Cyphomandra betacea* in adult male Sprague Dawley rats fed with high fat diet.

## 2. Materials and Methods

### 2.1. Materials

Freshly harvested Tamarillo (*Cyphomandra betacea*) was collected from Cameron Highland and stored at 4°C in Nutrition laboratory, Faculty of Medicine and Health Sciences, Universiti Putra Malaysia. Normal rat chow pallet was purchased from Saintik Enterprise (Malaysia). RANSOD kit (RANDOX Laboratory Ltd., USA), RANSEL kit (RANDOX Laboratories Ltd., USA), TAS kit (RANDOX Laboratories Ltd., Crumlin, UK), Rat TNF-*α* and IL-6 Platinum ELISA kit (eBioscience, USA), and test kits for measuring blood glucose (Sigma-Aldrich, USA) and lipid profile (Roche, Germany) were all supplied from Scientifacts Sdn. Bhd. (Malaysia).

### 2.2. Diet

The high fat diet was adapted based on the composition provided by Levin and Dunn-Meynell [19]. The high fat diet contains 414.0 kcal/100 g with 43% as carbohydrate, 17% as protein, and 40% as fat (Table 1). The diet consists of a mixture of 68% normal rat chow pellet (Saintik Enterprise, Malaysia), 20% instant milk powder (Dutch Lady), 6% corn oil (Krystal), and 6% ghee (Crispo). Meanwhile the normal rat chow diet contains 306.2 kcal/100 g with 48.8% as carbohydrate, 21% as protein, and 3% as fat. All ingredients of high fat diet were thoroughly mixed and baked in the oven at 65°C overnight. The high fat diet prepared was given to the obese induced rats for 10 weeks of obesity induction week. The normal rat chow diet was referred to as the control diet and given to negative control rats.

*C. betacea* was used as treatment extract in the study. Every sample was cleaned and washed to remove any residual compost by using tap water. These samples were cut into pieces and stored at −80°C. Then, the samples were freeze-dried to remove the moisture content. After freeze-drying, the dried sample was homogenously ground into fine powder by using dry grinder.* C. betacea* extract was given at three different doses: 150, 200, and 300 mg kg^−1^. The respective treatment extracts were prepared by adding 15 g, 20 g, and 30 g of freeze-dried* C. betacea* extract to 100 mL of distilled water and given to the* C. betacea* treatment group.

### 2.3. Animal Study Design

All experiment protocols were approved by the Institutional Animal Care and Use Committee, Universiti Putra Malaysia. Male Sprague Dawley rats (*n* = 40) at the age of 8 weeks, weighing between 200 and 250 g, were acclimatized for one week under room temperature (28 ± 2°C) with regular light and dark cycle and free access to food and water. Following acclimatization, the rats were randomly divided into five groups (*n* = 8). Four groups were given 25 g/per rat high fat diet for obesity induction week. Another group noted as Negative Control (NC) was given 25 g/per rat normal rat chow diet (Saintik Enterprise, Malaysia) throughout the study period.

After 10 weeks of obesity induction, the mean body weight and BMI of the high fat diet groups were compared to those of NC group. The group with significantly higher body weight and BMI were considered as obese [20] and subsequently received* C. betacea* extract or placebo treatment following treatment phase for 7 weeks. The high fat diet was continuously given to the obese induced rats during treatment period.

*C. betacea* supplement was given once daily to the* C. betacea* treated rats groups: Tamarillo low dosage group (150 mg kg^−1^) (TLDG), Tamarillo medium dosage group (200 mg kg^−1^) (TMDG), and Tamarillo high dosage group (300 mg kg^−1^) (THDG) via oral gavage (0.2 mL/100 g), while Negative Control group (NC) and Positive Control group (PC) received distilled water as placebo.

### 2.4. Analyses

#### 2.4.1. Blood Sampling

Approximately 3 mL blood was collected into EDTA and lithium heparin tubes via cardiac puncture. Intraperitoneal injection with zoletil (tiletamine 15 mg/kg and zolazepam 15 mg/kg) was performed on the rats to alleviate and minimize any pain. Then the blood collected in the EDTA tube was centrifuged at 3000 rpm at 4°C for 10 min and the plasma was stored at −80°C. The blood collected in the heparinised tube was processed in the same day for superoxide dismutase (SOD) and glutathione peroxidase (GPx) evaluation.

#### 2.4.2. Bodyweight, Food Intake, and BMI

Individual bodyweight was recorded weekly using electrical balance. The amount of leftover food was weighed daily using electrical balance and deducted from the amount of food given (25 g/per rat) in order to calculate daily food intake. Food intake was the amount of food (g) consumed by each rat within 24 hours. Meanwhile, the body length (cm) (nose-to-anus) was determined at week 7 to determine the BMI. BMI for normal adult rats ranged between 0.45 ± 0.02 and 0.68 ± 0.05 g/cm^2^ [20]. BMI was calculated using the following formula:

(1) $$\begin{array}{c} \text{Body mass index (BMI)}=\frac{\text{body weight (g)}}{\text{body lengt}h^{\text{2}}\left(\text{c}{\text{m}}^{\text{2}}\right)}. \end{array}$$

#### 2.4.3. Biochemical Test

Blood samples were taken from the overnight unfed rats. In this study, blood glucose was analysed by glucose oxidase (GO) assay kit (Sigma-Aldrich, USA), lipid profile (total cholesterol, triglyceride, LDL-C, and HDL-C) was evaluated by using a diagnostic reagent test kit (Roche, Germany) on a Hitachi Automatic Analyzer 902 (Tokyo, Japan). The activity of superoxide dismutase (SOD) was analysed by using a RANSOD kit (RANDOX Laboratory Ltd., USA); 0.5 mL heparinised whole blood sample was centrifuged at 3000 rpm for 10 min. The plasma was then aspirated off and later was washed four times with 3 mL normal saline (0.9% NaCl) solution for 10 min and followed by centrifugation at 3000 rpm after each wash. The washed centrifuged erythrocyte was made up to 2 mL with cold distilled water, mixed, and left to stand at 4°C for 15 min to form lysate. The absorbance was read spectrophotometrically at 505 nm via clinical chemistry analyzer machine (Vitalab Selectra E, Germany). Meanwhile, the activity of glutathione peroxidase (GPx) was analysed by using a RANSEL kit (RANDOX Laboratories Ltd., USA); 0.05 mL of heparinised whole blood sample was added with 2 mL of Ransel diluting agent. The sample was mixed well and incubated for 5 min. Absorbance was then read at 30 nm via clinical chemistry analyzer machine (Vitalab Selectra E, Germany). Total antioxidant status (TAS) was analysed by using a TAS kit (RANDOX Laboratories Ltd., Crumlin, UK); inflammatory biomarkers (TNF-*α* and IL-6) were analysed by using the Rat TNF-*α* and IL-6 Platinum ELISA kit (eBioscience, USA) which were all according to manufacturer's instructions, respectively.

### 2.5. Statistical Analysis

Data were expressed as mean ± standard deviation. Data were analysed by using one-way ANOVA using SPSS for windows version 21. Duncan's Multiple Range Test was used to test whether there were significant differences between the experimental groups. A significant difference is considered at the level of *p* < 0.05.

## 3. Result

### 3.1. Food Intake and Body Weight

All rats appeared to be healthy throughout the study and ate the food provided during the experimental period. After obesity induction week (week 10), there were no significant differences of food intake observed for all groups (*p* > 0.05). However, a significant difference can be seen for the caloric intake between the negative control group and obese induced groups (*p* < 0.05) (Table 2).

There were significant weight differences observed between the negative control group and all the obese induced groups at week 10 (*p* < 0.05) (Table 3). Similarly, there were also significant BMI differences found between the negative control group and all the obese induced groups (Table 3) (*p* < 0.05). Since all the obese induced groups had significantly higher mean bodyweight and BMI than the negative control group at week 10, they were all verified to be obese and therefore proceeded to the supplementation phase.

After treatment week (week 7), there was weight reduction detected in TMDG and THDG. THDG (462.50 ± 23.02 g) showed lowest bodyweight as compared to positive control group followed by TMDG (464.00 ± 47.15 g) and TLDG (482.17 ± 61.47 g), respectively (*p* > 0.05). There was also BMI reduction detected in all* C. betacea* treated groups. Lowest BMI can be seen in THDG (0.60 ± 0.08 g/cm^2^) followed by TMDG (0.66 ± 0.06 g/cm^2^) and TLDG (0.67 ± 0.11 g/cm^2^), respectively (*p* > 0.05) (Table 3).

### 3.2. Blood Glucose and Lipid Profile

After obesity induction week (week 10), there were significant increments of glucose, cholesterol, triglyceride, and LDL-C and significant decrement of HDL-C in the obese induced group as compared to negative control group (*p* < 0.05) (Table 4).

Following treatment week (week 7), there were significant decrements of cholesterol and significant HDL-C increment found in the* C. betacea* treated groups as compared to negative control and positive control group (*p* < 0.05). Although there was a positive reduction in triglyceride, LDL-C, and glucose found in the* C. betacea* treated groups, there was no significant difference detected between* C. betacea* treated groups with positive control group or negative control group (*p* > 0.05) (Table 4).

### 3.3. Antioxidant Enzyme

Treatment of obese rats with* C. betacea* extract led to positive increment of GPx and SOD activity (*p* > 0.05) and a significant increment in TAS level (*p* < 0.05) in* C. betacea* treated group as compared to negative control group and positive control group (Table 5). THDG rats showed highest value for all antioxidant enzymes activity (GPx, SOD, and TAS) followed by TMDG and TLDG, respectively.

### 3.4. Inflammatory Biomarkers

Supplementation of* C. betacea* extracts results in decrement of TNF-*α* concentration in* C. betacea* treated group. The lowest concentration can be seen in THDG (36.67 ± 44.57 pg/mL), followed by TMDG (43.33 ± 32.04 pg/mL) and TLDG (46.67 ± 39.33 pg/mL). There was a significant difference of TNF-*α* found in* C. betacea* treated group as compared with positive control group (*p* < 0.05) (Table 6).

Likewise, supplementation of* C. betacea* extracts also results in significant decrement of IL-6 concentration in* C. betacea* treated group. The lowest concentration can be seen in THDG (286.67 ± 306.38 pg/mL), followed by TMDG (380.00 ± 241.00 pg/mL) and TLDG (476.67 ± 524.66 pg/mL) (*p* < 0.05).

## 4. Discussion

Experimental obesity is usually taken as any significant increase in body weight or energy content relative to control animals [21]. In most studies, the degree of obesity has been evaluated by comparing body weight or fat of the experimental group fed with high fat diet or energy dense diet with control animals that show normal growth while being fed chow or low-fat diet [9, 10, 22]. There is a lack of information on anthropometrical parameters in laboratory rats. However, the method of verifying obese status via BMI was demonstrated in an obesity research conducted by Novelli et al. [20] and affirms that the BMI for normal adult rats ranged between 0.45 ± 0.02 and 0.68 ± 0.05 g/cm^2^.

During the obesity induction period, the daily food intake between groups showed no significant differences (*p* > 0.05); it was revealed that the amount of food intake was not affected by the calories content in normal chow diet and high fat diet. It was suggested that food intake was influenced by sensitivity of the rats to the palatability of food in the diet. Palatable food such as food high in fat and sugar inhibits the satiety signal and upregulates hunger sensation [11].

The continuous consumption of high fat diet results in significantly higher caloric intake compared to normal chow diet; therefore *p* < 0.05 showed positive increment of body weight thus leading to obese state. Previous literature demonstrated that a fat-rich diet induces obesity by increasing energy intake [10, 22–24]. Fat is the most energy dense nutrient (9 kcal/g versus 4 kcal/g for carbohydrate and protein); therefore, an addition of fat to food increases calories and also energy density [25].

After receiving their respective diet for 10 weeks, the obese induced rats gained significantly higher body weight and BMI than the negative control rats, thus verified to be obese. In this present study, the high fat diet consists of 40% of fat of total energy. Diet rich in fat induced obesity not only in human but also in animals. Previous literature mentioned a positive relationship between the level of fat in the diet and body weight or weight gain [24, 26–29]. Therefore, the relationship in human or animals models which have more dietary fat leading to greater obesity showed that the fat content of the diet is an important factor in energy balance. As a result, diets containing more than 30% of total energy as fat lead to the development of obesity [25].

An increase of glucose level can be seen following obesity induction. Previous study explained elevated blood glucose levels in animals consuming high fat diet; it was assumed that chronic exposure to elevated levels of fatty acids induces an increase in fatty acid oxidation and a decrease in glucose oxidation [30]. It was also reported that ingestion of high saturated fat induced an increase in hepatic liposynthetic gene expression and impairment in glucose metabolism, which can be seen through impaired translocation of glucose transporter-4 activity, suppressed expression of glucose transporter-4, inhibited expression of hepatic glycolytic and lipogenic enzymes, and impaired insulin signalling [31, 32].

As a result from high fat diet consumption, there were increments of TG, cholesterol, and LDL; meanwhile there was a decrease in HDL. It also can be seen that LDL and TG level were higher in obese induced rats compared to negative control group. LDL increases the rate of triacylglycerol catabolism by mobilizing fats from the liver to adipose tissue. It carries 60% to 70% of total cholesterol in the serum [33]. The hypertriglyceridemia condition resulting from high fat diet could be caused by enhanced liver VLDL-triglyceride secretion into circulation [34]. It was reported that, with unsaturated triglyceride, upregulating of LDL receptors occurred compared to saturated triglyceride. Therefore, it was suggested that dietary fatty acids can induced changes in cellular membrane lipids and may influence certain metabolic properties, such as receptor-mediated uptake of lipoproteins [35]. Meanwhile, the hypercholesteremia condition is associated with enhanced oxidizability of LDL molecules. The lag phase of lipid oxidation is shorter in LDL particles from obesity; rapid lipid peroxidation in the polyunsaturated fatty acids of LDL particles subsequently occurs [3, 36]. The susceptibility of lipids to oxidative modification is also shown by higher concentrations of 4-HNE per unit intramuscular triglycerides in obesity [37].

In this study, the antioxidants enzyme activities of GPx, SOD, and TAS were lower in the positive control group. Decrease of antioxidant enzyme may be related to rapid consumption and exhaustion of storage of this enzyme in combating free radicals generated during development of obesity. In addition activities of major antioxidant enzymes may also be inadequate in obesity [38].

Bełtowski et al. [39] demonstrated that activity of erythrocyte SOD and GPx activities were lower by 29–42% in the high fat, high calorie fed animals as compared to control animals after diet period. Similar lower result was also found for TAS value. At the early stage of obesity, it has been proposed that there may be an initial elevation in antioxidant enzymes to counteract oxidative stress; as chronic obesity developed, it continually depletes the sources of antioxidant enzymes [40, 41]. Meanwhile, TAS has been used as comprehensive measure of radical-squelching capacity by antioxidant in plasma. Lower TAS value was directly related to lower levels of various forms of plasma carotenoids such as *α*-carotene, *β*-carotene and *α*-tocopherol, *β*-tocopherol, and *ϒ*-tocopherol [41].

The effect of high fat diet on proinflammatory cytokines can be seen with the increment of IL-6 and TNF-*α* in positive control group. Numerous previous studies found that, compared to healthy lean individuals, overweight and obese individuals have higher proinflammatory cytokines [42, 43]. Obesity is characterized by having a greater number of adipose tissues (hyperplasia) and an increase in the size of adipocytes (hypertrophy) [44, 45]. These conditions lead to oxygen depletion in adipose tissue, hence causing adipocyte cell death. In addition, the excess storage of triacylglycerol (TAG) from dietary intake results in an excessive influx of free fatty acids into blood circulation. Therefore, taken together, this condition can lead to low-grade inflammation characterized by the overproduction of proinflammatory adipocytokines [46].

The supplementation of* C. betacea* demonstrated that the treatment groups showed lower bodyweight as compared to positive control group. This finding supports* C. betacea* ability in maintaining the current bodyweight. Lister et al. [14] stated that the amount of water for red and yellow* C. betacea* was 86.06 g and 86.30 g, respectively. Meanwhile the value of fibre was stated to be 3.3 g. As such,* C. betacea* showed high water content and considerable amount of fibre. The rationale behind the potential role of an increased consumption of fruit in the prevention of overweight and obesity is related to several features of whole fruit which include high water content, low energy density, and high content of dietary fibres, of which viscous dietary fibres constitute a considerable proportion. Dietary fibre and, in particular, viscous dietary fibre, which present in fruit in considerable amounts, have been shown to increase postprandial satiety and to decrease subsequent hunger in short-term studies [12].

Administering* C. betacea* extract to the high fat diet rats showed positive increment of antioxidants enzyme.* C. betacea* has been demonstrated to have antioxidant activity and contained phenolic composition [14–18]. Dietary nutrients and specific foods, rich in polyphenols (e.g., anthocyanin, flavonoid), could play an important role in the prevention and control of complication arising from oxidative stress, through its role in increasing the circulation of antioxidant compounds [47, 48]. In addition, polyphenols are capable of neutralizing reactive species, due to their favourable number and position of hydroxyls [49–51]. As reactive oxygen species (ROS) decrease the expression of adiponectin, treatment with antioxidant or ROS inhibitor could restore the regulation of adipokines. Thus, supplementation with antioxidant would reduce the risk of complication related to obesity and oxidative stress [52].

*C. betacea* also successfully decrease the inflammatory biomarkers in the* C. betacea* treated groups. Previous study showed that carotenoids in fruit and vegetable were associated with anti-inflammatory effects [53]. The biological mechanisms for the protective effect of greater variety in fruit and vegetable intake are not entirely clear but may be attributed to the singular or synergistic action of several bioactive compounds. Mounting evidence shows that bioactive compounds such as carotenoids, flavonoids, phytoestrogens, dietary fibre, and resveratrol, which are present in a wide variety of fruits and vegetables, influence the risk of oxidative stress [54, 55]. It was also reported that low dietary intake of fruits and vegetables and low serum concentration of flavonoids, carotenoids, and vitamin C, which may indirectly represent low fruit and vegetable variety, have been associated with increased inflammatory status [56–59].

Throughout the treatment period, highest dose of oral administration of* C. betacea* (300 mg kg^−1^) did not induce mortality. There were no toxic signs such as vomiting, fur loss, diarrhoea, or death detected in* C. betacea* treated group; therefore, it could be considered safe [60].

## 5. Conclusion

Overall, treatment of obese induced rats with* C. betacea* extract showed its potential in weight maintenance and positive lipid lowering effect and demonstrated an increment of antioxidant activity of SOD, GPx, and TAS and exhibited anti-inflammatory effects as showed in the decrements of inflammatory biomarkers. Therefore, consumption of* C. betacea* in daily dietary intake is a one-step action towards prevention of obesity and weight management principle.

## Figures and Tables

**Table 1:** Composition of high fat diet and normal rat chow diet.

High fat diet		Normal rat chow diet	
**Nutrients**	**%/100 g**	**Nutrients**	**%/100 g**
Carbohydrate	43	Carbohydrate	48.8
Protein	17	Protein	21
Fat	40	Fat	3
**Ingredients**	**g/100 g**	Calcium	0.8
Powdered rat feed	68.0	Phosphorus	0.4
Maize oil	6.0	Fiber	5
Ghee	6.0	Moisture	13
Milk powder	20.0	Ash	8
Total energy (kcal/100 g)	414.0	Total energy (kcal/100 g)	306.2

**Table 2:** Mean of food and caloric intake of rats after the obesity induction week (week 10) and after treatment week (week 7).

Group	Food intake (g)		Caloric intake (kcal)	
	Initial (week 10)	Final (week 7)	Initial (week 10)	Final (week 7)
NC	18.39 ± 0.50	19.41 ± 2.12	69.89 ± 1.91^a^	73.75 ± 8.06^a^
PC	18.48 ± 1.04	21.57 ± 0.59	80.66 ± 4.53^b^	94.44 ± 2.60^b^
TLDG	18.61 ± 1.93	21.30 ± 0.88	81.48 ± 8.45^b^	94.93 ± 4.04^b^
TMDG	19.35 ± 2.18	21.78 ± 1.11	84.74 ± 9.57^b^	96.88 ± 4.75^b^
THDG	19.15 ± 1.65	21.61 ± 1.35	83.85 ± 7.09^b^	96.13 ± 5.90^b^

Values represent the Mean ± SD (*n* = 8). ^a^ *p* < 0.05 versus positive control group; ^b^ *p* < 0.05 versus negative control group. NC: negative control; PC: positive control; TLDG: *C*. *betacea* extract 150 mg/kg; TMDG: *C*. *betacea* extract 200 mg/kg; THDG: *C*. *betacea* extract 300 mg/kg.

**Table 3:** Mean of body weight and body mass index (BMI) of rats after the obesity induction week (week 10) and after treatment week (week 7).

Group	Bodyweight (g)		Body mass index (BMI) (g/cm^2^)	
	Initial (week 10)	Final (week 7)	Initial (week 10)	Final (week 7)
NC	413.83 ± 40.46^a^	453.17 ± 48.22	0.56 ± 0.05^a^	0.62 ± 0.05
PC	465.83 ± 22.33^b^	508.00 ± 56.06	0.73 ± 0.05^b^	0.71 ± 0.08
TLDG	471.33 ± 40.54^b^	482.17 ± 61.47	0.71 ± 0.04^b^	0.67 ± 0.11
TMDG	468.50 ± 53.64^b^	464.00 ± 47.15	0.71 ± 0.05^b^	0.66 ± 0.06
THDG	470.50 ± 47.75^b^	462.50 ± 23.02	0.72 ± 0.08^b^	0.60 ± 0.08

Values represent the Mean ± SD (*n* = 8). ^a^ *p* < 0.05 versus positive control group; ^b^ *p* < 0.05 versus negative control group. NC: negative control; PC: positive control; TLDG: *C*. *betacea* extract 150 mg/kg; TMDG: *C*. *betacea* extract 200 mg/kg; THDG: *C*. *betacea* extract 300 mg/kg.

**Table 4:** Mean of blood glucose and lipid profile of rats after obesity induction (week 10) and after treatment (week 7).

	Group				
	NC	PC	TLDG	TMDG	THDG
Blood glucose					
(week 10)	6.00 ± 0.80^a^	7.47 ± 0.69^b^	7.95 ± 0.88^b^	7.45 ± 1.32^b^	7.72 ± 1.8^b^
(week 7)	6.95 ± 0.99	7.07 ± 0.69	6.80 ± 0.88	6.30 ± 0.63	5.97 ± 0.21
TC					
(week 10)	1.65 ± 0.52^a^	2.63 ± 0.67^b^	1.84 ± 0.07^b^	1.85 ± 0.10^b^	1.79 ± 0.49^b^
(week 7)	1.35 ± 0.31^a^	1.86 ± 0.43^b^	1.79 ± 0.37^ab^	1.75 ± 0.25^ab^	1.75 ± 0.22^ab^
TG					
(week 10)	0.51 ± 0.13^a^	0.88 ± 0.30^b^	0.81 ± 0.07^b^	0.75 ± 0.08^b^	0.82 ± 0.12^b^
(week 7)	0.51 ± 0.29	0.52 ± 0.09	0.50 ± 0.13	0.48 ± 0.15	0.46 ± 0.10
LDL-C					
(week 10)	0.19 ± 0.17^a^	0.72 ± 1.14^b^	0.25 ± 0.16^b^	0.23 ± 0.12^b^	0.27 ± 0.15^b^
(week 7)	0.16 ± 0.13	0.19 ± 0.10	0.17 ± 0.15	0.18 ± 0.04	0.18 ± 0.10
HDL-C					
(week 10)	1.33 ± 0.39^a^	0.89 ± 0.62^b^	1.09 ± 0.36^b^	0.89 ± 0.12^b^	0.92 ± 0.21^b^
(week 7)	0.98 ± 0.33^a^	1.18 ± 0.06^b^	1.40 ± 0.16^ab^	1.45 ± 0.22^ab^	1.55 ± 0.36^ab^

Values represent the Mean ± SD (*n* = 8). ^a^ *p* < 0.05 versus positive control group; ^b^ *p* < 0.05 versus negative control group. NC: negative control; PC: positive control; TLDG: *C*. *betacea* extract 150 mg/kg; TMDG: *C*. *betacea* extract 200 mg/kg; THDG: *C*. *betacea* extract 300 mg/kg.

**Table 5:** Effect *C. betacea* extract on antioxidant enzyme activities.

Group	Antioxidant enzyme activities		
	GPx (UL^−1^)	SOD (UmL^−1^)	TAS (mmolL^−1^)
NC	1798.00 ± 2.26	7.15 ± 0.96	1.20 ± 0.11
PC	1735.02 ± 2.43	7.11 ± 1.01	1.47 ± 0.10
TLDG	2195.95 ± 5.33	7.61 ± 0.13	1.78 ± 0.16^ab^
TMDG	2298.90 ± 3.01	7.62 ± 0.13	1.87 ± 0.19^ab^
THDG	2422.07 ± 5.76^ab^	7.76 ± 0.13	1.97 ± 0.48^ab^

Values represent the Mean ± SD (*n* = 8). ^a^ *p* < 0.05 versus positive control group; ^b^ *p* < 0.05 versus negative control group. NC: negative control; PC: positive control; TLDG: *C*. *betacea* extract 150 mg/kg; TMDG: *C*. *betacea* extract 200 mg/kg; THDG: *C*. *betacea* extract 300 mg/kg.

**Table 6:** Effect *C*. *betacea* extract on inflammatory biomarkers.

Groups					
	NC	PC	TLDG	TMDG	THDG
TNF-*α* (pg/mL)	50.00 ± 3.52^a^	113.33 ± 7.34^b^	46.67 ± 3.93^a^	43.33 ± 3.20^a^	36.67 ± 4.46^a^
IL-6 (pg/mL)	660.00 ± 3.66	1066.67 ± 5.71	476.67 ± 5.25^a^	380.00 ± 2.41^a^	286.67 ± 3.06^a^

Values represent the Mean ± SD (*n* = 8). ^a^ *p* < 0.05 versus positive control group; ^b^ *p* < 0.05 versus negative control group. NC: negative control; PC: positive control; TLDG: *C*. *betacea* extract 150 mg/kg; TMDG: *C*. *betacea* extract 200 mg/kg; THDG: *C*. *betacea* extract 300 mg/kg.
